# Effects of gymnastics exercises on executive function in children with autism spectrum disorder aged 6 to 9 years: a pilot study of a randomized controlled trial

**DOI:** 10.3389/fpsyg.2025.1660305

**Published:** 2025-10-27

**Authors:** Chenliang Deng, Zhimin Zhu, Ganglin Luo, Huan Zeng, Xinwen Jiang, Qiaoyan Yu, Xiaofei Pan

**Affiliations:** ^1^School of Physical Education, Chengdu Sport University, Chengdu, China; ^2^Sports Department, University of Electronic Science and Technology of China, Chengdu, China; ^3^Department of Military Education and Training, Police Academy of the Armed Police, Chengdu, China; ^4^Chengdu Chenghua District Special Education School, Chengdu, China

**Keywords:** children with autism spectrum disorder, executive function, gymnastics exercises, inhibitory control, working memory, cognitive flexibility

## Abstract

**Objective:**

To explore the improvement effects of 12-week gymnastics exercises on executive function (including inhibitory control, working memory, and cognitive flexibility) in children aged 6 to 9 years with autism spectrum disorder (ASD), and to provide a basis for motor interventions in ASD children.

**Study design:**

A randomized controlled trial (RCT) design was adopted.

**Methods:**

Twenty-four ASD children aged 6–9 years from a special school in Chenghua District, Chengdu, were selected and randomly divided into an experimental group (12 cases) and a control group (12 cases). The experimental group received gymnastics exercises (covering movements such as walking, running, crawling, tumbling, and jumping) 3 times a week for 40 min each time, while the control group maintained routine daily activities. Before and after the intervention, the day-night Stroop task, self-ordered pointing task, and Wisconsin card sorting test were used to assess inhibitory control, working memory, and cognitive flexibility, respectively. Independent samples t-tests and paired samples t-tests were performed using SPSS 29.0 to analyze intergroup and intragroup differences.

**Results:**

After the intervention, the scores of inhibitory control and working memory in the experimental group were significantly higher than those before the intervention (both *p* < 0.01), and the score of cognitive flexibility was also significantly improved (*p* < 0.05). The control group showed a significant difference in working memory (p < 0.05), while there were no statistically significant differences in inhibitory control and cognitive flexibility before and after the intervention (*p* > 0.05). Intergroup comparison showed that the improvement in each dimension of executive function in the experimental group was better than that in the control group, but there was no significant difference (*p* > 0.05), which may be related to the short intervention time.

**Conclusion:**

12-week gymnastics exercises can effectively improve the executive function of 6-9-year-old children with ASD, and can be used as an effective motor intervention for ASD children.

## 1 Introduction

Autism spectrum disorder (ASD) is a neurodevelopmental disorder characterized by core features of social communication difficulties, restricted interests, and repetitive stereotyped behaviors (Tao, 1982; Lord et al., 2018; Sun et al., 2018; Kelly et al., 2021; Davoudi et al., 2023), with a remarkably increasing global prevalence. World Health Organization (WHO) data show that the global prevalence of ASD in children is approximately 1 in 100 (Gesundheit et al., 2025). The latest 2023 report from the U. S. Centers for Disease Control and Prevention (CDC) further indicates that 1 in every 36 children in the United States is diagnosed with ASD, accounting for 2.8%. In China, the total number of ASD patients exceeds 13 million, including approximately 2 million children aged 0–14, with a prevalence rate of about 1% (Wucailu Autism Research Institute, 2024; Liu, 2025). This population not only faces multiple cognitive and behavioral challenges but also imposes heavy psychological and economic burdens on their families (Lu et al., 2015). Therefore, exploring safe and effective interventions to improve the functional outcomes of children with ASD has become a critical public health issue.

Executive function deficits represent one of the core cognitive characteristics in children with ASD. As the regulatory core of higher-order cognitive abilities, EF encompasses three key components: inhibitory control, working memory, and cognitive flexibility. Their synergistic effects directly influence an individual’s task switching, impulse regulation, and adaptive behavior (Diamond, 2013; Demetriou et al., 2018). Specifically: Inhibitory control regulates interference from irrelevant stimuli and suppression of dominant responses, including two dimensions: response inhibition (e.g., restraining immediate action impulses when following the “stop at red light” rule) and interference inhibition (e.g., ignoring external distractions to focus in class; Li and Zhang, 2020). Children with ASD commonly exhibit deficits in this ability, manifesting as frequent impulsive behaviors and difficulty filtering irrelevant information, which exacerbates social difficulties and stereotyped behaviors (An et al., 2019a; Davoudi et al., 2023). Working memory, as the core for temporary information storage and processing, is crucial for learning, problem-solving, and social interaction (Li et al., 2004). Children with ASD often show shorter information retention and lower processing efficiency in working memory—for example, struggling to quickly process dynamic social information (e.g., others’ facial expressions and language), thereby hindering interactive responses (Li, 2014; An et al., 2019b; Liu, 2023). Cognitive flexibility refers to the ability to flexibly adjust thinking strategies and behavioral patterns according to environmental changes (Liang, 2014). Deficits in this ability in children with ASD lead to increased stereotyped behaviors and social adaptation difficulties, which are closely associated with abnormal functional connectivity in brain regions such as the prefrontal cortex, cerebellum, and parietal lobe (Chen et al., 2024).

Motor intervention, as a non-invasive approach to improving executive function, has been widely validated in interventions for children with ASD. For instance, 12-week youth basketball training can enhance executive function by altering functional connectivity in brain regions such as the prefrontal lobe and inferior temporal gyrus (Wang, 2020); sports games can improve cognitive and behavioral performance in young children with ASD (Kou and Fan, 2020); youth fun track-and-field combined with ball games and sports games have, respectively, been confirmed to enhance cognitive flexibility and cognitive shifting abilities in children with ASD (Yan, 2023; Li, 2022). However, existing studies mainly focus on single forms such as ball games or aerobic exercises, while systematic explorations of gymnastics exercises remain scarce. Based on the motor-cognitive integration theory, complex motor tasks can simultaneously improve motor and cognitive functions by activating the prefrontal-cerebellar neural network (Gomez-Pinilla and Hillman, 2013; Hillman et al., 2014, 2020). As a comprehensive sport integrating strength, balance, and coordination, gymnastics movements (e.g., tumbling, directional jumping, obstacle crawling) require multisensory integration of vision, proprioception, etc., and feature action sequencing and immediate feedback. Compared with the repetitive actions of ball games or the single rhythm of aerobic exercises, these characteristics better meet the sensory integration needs of children with ASD, not only developing their body control and coordination (Yang, 2016) but also potentially exerting unique improvements on executive function (Jing, 2001; Zhang, 2023). Preliminary studies have found that basic gymnastics can enhance memory and cognitive comprehension in preschool children with autism (Shao, 2014), but no study has systematically explored the overall impact of gymnastics exercises on the three core components of executive function in children with ASD.

From a developmental perspective, 6–9 years old is a critical period for children’s executive function development, during which neuroplasticity is high, making interventions more likely to produce significant and lasting effects (Ozonoff and Strayer, 1997; Hou and Yang, 2016). However, current motor intervention studies for children with ASD in this age group mostly focus on single executive function components, lacking systematic assessment of inhibitory control, working memory, and cognitive flexibility. Meanwhile, although the comprehensive advantages of gymnastics have been preliminarily recognized, no randomized controlled study has verified its overall improvement effect on executive function in children with ASD. Therefore, this randomized controlled trial (RCT) aimed to systematically investigate the effects of 12-week structured gymnastics exercises on the three core components of executive function (inhibitory control, working memory, cognitive flexibility) in 6–9-year-old children with ASD, with the goal of providing a theoretical basis and actionable practical strategies for personalized motor interventions in children with ASD.

## 2 Methods

### 2.1 Study participants

Twenty-four children aged 6–9 years with ASD were recruited from a special school in Chenghua District, Chengdu. All children met the diagnostic criteria for ASD in the Diagnostic and Statistical Manual of Mental Disorders, Fifth Edition (DSM-5) (American Psychiatric Association, 2013), and satisfied the following inclusion and exclusion criteria: (1) Inclusion criteria: ① Diagnosed with mild to moderate ASD by professional medical institutions (e.g., developmental behavior departments of children’s hospitals, professional autism rehabilitation centers); ② Able to independently complete 40-min motor tasks; ③ Without severe physical diseases (e.g., heart disease, skeletal deformities) or other mental disorders (e.g., schizophrenia); ④ Guardians signed the informed consent form and committed to cooperating throughout the study. (2) Exclusion criteria: ① Comorbid visual/hearing impairments or requiring assistive devices to perform basic movements (e.g., sitting, standing); ② Receiving other cognitive trainings (e.g., attention training programs) or motor interventions (e.g., specialized ball game training); ③ Having neurological diseases (e.g., epilepsy) or being in a stage of pharmacotherapy affecting cognitive function.

Participants were grouped using a random number table method. An independent statistician not involved in data collection generated random sequences via SPSS 29.0, and allocation concealment was implemented using sealed opaque envelopes (kept by a third party and opened to inform researchers after grouping). Eventually, 24 children were divided into an experimental group and a control group, with 12 participants in each group. Due to the significant differences in intervention forms (structured gymnastics vs. routine activities), blinding of participants and coaches was not feasible, but outcome assessors were blinded to group allocation to reduce measurement bias.

There were no significant differences in baseline data between the two groups, including gender (experimental group: male/female = 6/6; control group: male/female = 6/6), age, height, weight, and executive function (*p*>0.05), indicating comparability (Table 1).

**Table 1:** Comparison of baseline data between two groups of children with ASD (*M* ± SD).

Index	Group		*t*	*p*
	Experimental	Control		
Sex (Male/Female)	6/6	6/6	—	1.000
Age (yrs)	7.75 ± 1.14	7.92 ± 1.16	−0.355	0.726
Height (cm)	131.33 ± 9.74	131.24 ± 12.60	0.018	0.986
Weight (kg)	28.73 ± 8.60	29.94 ± 11.57	−0.297	0.769
Inhibitory Control	6.25 ± 1.29	6.58 ± 1.08	−0.686	0.500
Working Memory	21.50 ± 4.17	20.58 ± 4.10	0.543	0.592
Cognitive Flexibility	4.83 ± 1.53	4.83 ± 1.75	0.000	1.000

*p* > 0.05 indicates no significant difference; *p* < 0.05 indicates statistical significance; *p* < 0.01 indicates high statistical significance; the same below.

### 2.2 Experimental design

This study was a RCT designed and implemented following the CONSORT 2010 guidelines. The experimental period was from September to December 2024, and the intervention was conducted in the sports venue of a special school in Chenghua District, Chengdu.

#### 2.2.1 Experimental group

Received 12-week structured gymnastics exercises, 3 times per week, 40 min each time.

Control group: Engaged in routine campus activities (non-overlapping with the experimental group to ensure independence), with activity forms and frequency matched to the experimental group (3 times per week, 40 min each time).

Executive function (inhibitory control, working memory, cognitive flexibility) was assessed in both groups 1 week before the intervention (T₀) and 1 week after the intervention (T₁). All assessments were conducted by the same principal investigator blinded to group allocation to reduce measurement errors. During the intervention, no children withdrew, and no adverse events (e.g., sports injuries, emotional outbursts) occurred in either group.

### 2.3 Intervention protocols

#### 2.3.1 Experimental group: structured gymnastics exercises

The gymnastics intervention protocol was based on the Toddler Gymnastics Tutorial (Wen, 2012) and the Compulsory Education Life Adaptation Curriculum Standards for Intellectually Disabled Schools (2016 Edition) (Ministry of Education of the People’s Republic of China, 2018). Combining the motor development characteristics of children with ASD and expert interview opinions (three special education experts and two gymnastics coaches were invited for semi-structured interviews to determine movement difficulty and safety), a progressive intervention model was adopted, covering four core movement categories: walking/running, crawling, tumbling, and jumping (Table 2).

**Table 2:** Statistics of four core movement categories.

Movement type	Specific content	Difficulty progression strategies
Walking/Running	Straight/curved walking, obstacle running (around marker cones), balance beam walking (with 90°/180° turns)	From “eyes open + slow speed” to “eyes closed + fast speed”; from “unidirectional movement” to “multidirectional changes”
Crawling	Hands-knees crawling, elbows-knees crawling, “L-shaped”/"S-shaped” route crawling	From “straight crawling” to “complex route crawling”; from “ipsilateral limb synchronization” to “contralateral limb alternation”
Tumbling	Straight body side roll, flexed body forward roll (with chest-hugging/lateral horizontal arm positions)	From “assisted completion” to “independent completion”; from “short-distance tumbling” to “long-distance continuous tumbling”
Jumping	Legs-together jump, jumping jacks, directional jump (adjusted according to marker cone direction), trampoline turning jump	From “stationary jumping” to “moving jumping”; from “low-intensity repetition” to “moderate-intensity combination”

A single training session consisted of three parts. Warm-up stage (5 min): Included jogging (3 laps around the venue, speed from slow to fast) + joint activities (neck, shoulder, wrist/ankle rotations) to warm up the children’s bodies. Main practice stage (30 min): 2–3 movement categories were selected for combined training, 10 min per category, with full assistance from two teachers experienced in special education and gymnastics instruction to ensure movement safety and standardization (e.g., protecting the neck during tumbling, assisting with balance during balance beam walking). Relaxation stage (5 min): Included static stretching (e.g., front thigh and back stretching) to help children relieve muscle tension.

Exercise intensity was controlled at a moderate level (average heart rate 100–120 beats/min), recorded in real time by teaching assistants using heart rate bracelets.

#### 2.3.2 Control group: routine campus activities

The control group’s activities were unstructured physical activities routinely arranged by the school, significantly differentiated from the experimental group’s structured gymnastics training in terms of movement complexity, goal targeting, and instructional professionalism, including: Free play (30 min daily): Children independently chose toys (building blocks, balls, slides, etc.) for unorganized play in indoor activity areas, with teachers only responsible for safety supervision without any targeted movement guidance. Simple physical training (3 times per week, 20 min each time): Included basic limb activities such as line walking, marching in place, and simple stretching (chest expansion, bending), with single-action design and no progressive difficulty, involving no complex coordination, balance, or rule-based training, and exercise intensity maintained at low to moderate levels (average heart rate 80–100 beats/min).

#### 2.3.3 Intervention fidelity control

Intervention implementation in both groups was recorded through “activity logs”: the experimental group recorded the number of attendees each time (to calculate attendance rate) and movement compliance rate (defined as independently completing 80% of movements); the control group recorded daily activity participation duration and the frequency of children’s autonomous activity choices. Final statistics showed an attendance rate of 92.3% and a movement compliance rate of 88.5% in the experimental group, and an activity participation rate of 90.7% in the control group, ensuring intervention fidelity.

### 2.4 Assessment tools

Executive function includes three core components: inhibitory control, working memory, and cognitive flexibility. This study used targeted tools to assess each component, all of which have good reliability and validity in children with ASD.

#### 2.4.1 Inhibitory control: day-night Stroop task

The day-night Stroop task (DNST) is a classic tool for assessing children’s inhibitory control (Montgomery and Koeltzow, 2010), requiring children to inhibit the automatic association of “sun→day” and “moon→night” and state the opposite (saying “night” when seeing the sun and “day” when seeing the moon). To control for potential biases in verbal comprehension among children with ASD, a picture matching task was conducted before assessment to confirm children’s conceptual understanding of “sun-day” and “moon-night” (100% accuracy required).

Materials: 8 white sun cards and 8 black moon cards.

Procedure: Divided into pre-training and formal testing. Pre-training ensured children understood the correspondence between “sun-day” and “moon-night.” In formal testing, cards were presented one by one, requiring children to give the opposite response within 3 s. The number of correct responses was recorded, with a total score range of 0–16 (higher scores indicating stronger inhibitory control).

Reliability and validity: This study supplemented the test–retest reliability for the ASD sample, showing an intraclass correlation coefficient (ICC) of 0.85 (95% CI: 0.62–0.94), indicating good stability in children with ASD.

Assessment environment and procedure: Conducted in a quiet room (environmental noise <40 dB) with dim lighting (illuminance ~100lux) on a standard Table (75 cm height). Pre-assessment practice instructions: “You will see a sun or moon card. If it’s the sun, say ‘night’; if it’s the moon, say ‘day’. Let us practice first.” During formal testing, the principal investigator used standardized instructions: “When you see the sun, say ‘night’; when you see the moon, say ‘day’. Try to be quick and accurate!” Assessments were completed by two professionally trained teachers, once at T₀ and once at T₁, each fixed at 10:00 a.m. to reduce circadian rhythm effects on cognitive performance.

#### 2.4.2 Working memory: self-ordered pointing task

The self-ordered pointing task (SOPT) is a typical paradigm for studying working memory (Sallum et al., 2017; Faja and Darling, 2019), requiring children to continuously select previously unselected cards after sequence changes, testing temporary information storage and processing abilities.

Materials: 5 cards with different patterns (sunflower, kitten, carrot, tanghulu, and eggplant).

Procedure: Divided into pre-training and formal testing. Pre-training used “2-card unselected selection” practice (first select 1 card, then select the unselected one after reordering) to ensure children understood the rules. Formal testing started with 2 cards, increasing by 1 card per round (up to 5) if selected correctly. Cards were randomly shuffled each round, and children were required to continuously select unselected cards. The task was terminated if the child made repeated errors in a card set.

Scoring criteria: 1 point for correct selection of 2 cards, 2 points for 3 cards, 3 points for 4 cards, 4 points for 5 cards. A total of 20 tests were conducted, with a full score of 80.

Assessment environment and procedure: Individually administered in a quiet room by two principal investigators using standardized instructions, with each child’s testing duration approximately 15 min.

#### 2.4.3 Cognitive flexibility: adapted Wisconsin card sorting test

Originally used to assess adult abstract thinking and flexible switching abilities, the adapted Wisconsin card sorting test (WCST) is suitable for children aged 6 and above (Crawley et al., 2020; Arango-Lasprilla et al., 2017), requiring children to flexibly classify cards based on rules of “shape, color, quantity, and presence of borders”.

Materials: 6 target cards with patterns: “1 red triangle,” “2 yellow triangles,” “1 green circle,” “2 green pentagrams,” “2 red circles (with black borders),” “1 yellow pentagram (with black borders),” covering 4 features (shape, color, quantity, presence of borders).

Procedure: Children were sequentially required to classify cards according to the rules of “shape→color→quantity→presence of borders,” with each rule used only once. The principal investigator provided timely “correct” or “wrong” feedback. Task instructions: “From the following 6 cards, select any 2 cards according to the rules of color, quantity, shape, and presence of borders. Each rule requires selecting only 2 cards.”

Scoring criteria: 0 points for all classification errors; 3 points for correct color-based classification, 3 points for correct shape-based classification, 2 points for correct quantity-based classification, 2 points for correct border-based classification, with a maximum total score of 10.

Assessment environment and procedure: Administered individually in a quiet room by two systematically trained special education teachers. Standardized instruction explanations and practice trials were conducted before testing to ensure children understood task requirements. Testing order between participants used a balanced design, and T₀ and T₁ tests for the same participant were completed by two principal investigators.

### 2.5 Data statistical analysis

Data were analyzed using SPSS 29.0 software: Measurement data were expressed as “mean ± standard deviation (M ± SD).” Paired samples t-tests were used for within-group comparisons before and after intervention, and independent samples t-tests were used for between-group comparisons. The test level was *α* = 0.05, with *p* < 0.05 indicating statistical significance and *p* < 0.01 indicating high statistical significance.

## 3 Results

This study systematically evaluated the improvement effects of 12-week gymnastics exercises on executive function (inhibitory control, working memory, cognitive flexibility) in 6–9-year-old children with ASD through independent samples t-tests for between-group differences and paired samples t-tests for within-group differences before and after intervention.

### 3.1 Intervention effects on inhibitory control

Inhibitory control was assessed using the DNST (total score 0–16, higher scores indicating stronger inhibitory control). The comparison of inhibitory control scores between the two groups before and after intervention is shown in Table 3.

**Table 3:** Comparison of inhibitory control scores between two groups of children with ASD before and after intervention (M ± SD).

Group	*N*	Before Intervention	After Intervention	*t*	*p*
Experimental group	12	6.25 ± 1.29	8.08 ± 1.68	−3.527	0.005
Control group	12	6.58 ± 1.08	7.17 ± 1.27	−1.465	0.171
*t*		−0.686	1.511		
*p*		0.500	0.145		

#### 3.1.1 Between-group differences

Baseline before intervention: The inhibitory control score of the experimental group was 6.25 ± 1.29, and that of the control group was 6.58 ± 1.08. Independent samples t-test showed no significant between-group difference (*t* = −0.686, *p* = 0.500>0.05), indicating that the two groups had comparable inhibitory control abilities at baseline.

Post-intervention endpoint: The inhibitory control score of the experimental group increased to 8.08 ± 1.68, and that of the control group was 7.17 ± 1.27. The experimental group scored higher than the control group, but the between-group difference still did not reach statistical significance (*t* = 1.511, *p* = 0.145>0.05).

#### 3.1.2 Within-group differences

Experimental group: The post-intervention inhibitory control score (8.08 ± 1.68) was significantly higher than the pre-intervention score (6.25 ± 1.29), with high statistical significance (*t* = −3.527, *p* = 0.005<0.01), with an average increase of 1.83 points. This indicates that 12-week gymnastics exercises can significantly enhance the inhibitory control ability of children in the experimental group.

Control group: The pre-intervention score was 6.58 ± 1.08, and the post-intervention score was 7.17 ± 1.27, with a small increase (average 0.59 points), but the difference was not statistically significant (*t* = −1.465, *p* = 0.171>0.05), suggesting that routine campus activities cannot effectively improve the inhibitory control of children with ASD.

### 3.2 Intervention effects on working memory

Working memory was assessed using the SOPT (total score 0–80, higher scores indicating stronger working memory). The comparison of working memory scores between the two groups before and after intervention is shown in Table 4.

**Table 4:** Comparison of working memory scores between two groups of children with ASD before and after intervention (M ± SD).

Group	*N*	Pre-intervention	Post-intervention	*t*	*p*
Experimental	12	21.50 ± 4.17	32.83 ± 6.03	−6.107	0.000
Control	12	20.58 ± 4.10	27.75 ± 6.81	−2.966	0.013
*t*		0.543	1.936		
*p*		0.592	0.066		

#### 3.2.1 Between-group differences

Baseline before intervention: The working memory score of the experimental group was 21.50 ± 4.17, and that of the control group was 20.58 ± 4.10. There was no significant between-group difference (*t* = 0.543, *p* = 0.592>0.05), indicating consistent baseline working memory levels between the two groups.

Post-intervention endpoint: The working memory score of the experimental group increased to 32.83 ± 6.03, and that of the control group was 27.75 ± 6.81. The experimental group scored higher than the control group, and the between-group difference approached statistical significance (*t* = 1.936, *p* = 0.066), suggesting that gymnastics exercises showed a better improvement trend in working memory than routine activities.

#### 3.2.2 Within-group differences

Experimental group: The post-intervention working memory score (32.83 ± 6.03) significantly increased compared with the pre-intervention score (21.50 ± 4.17), with an average increase of 11.33 points and high statistical significance (*t* = −6.107, P<0.001), indicating that gymnastics exercises can greatly enhance the working memory ability of children with ASD.

Control group: The post-intervention working memory score (27.75 ± 6.81) increased by 7.17 points compared with the pre-intervention score (20.58 ± 4.10), with statistical significance (*t* = −2.966, *p* = 0.013<0.05). It is speculated that this may be related to simple cognitive stimulation in routine activities (e.g., rule understanding in free play), but the improvement range was only 63.3% of that in the experimental group.

### 3.3 Intervention effects on cognitive flexibility

Cognitive flexibility was assessed using the adapted WCST (total score 0–10, higher scores indicating stronger cognitive flexibility). The comparison of cognitive flexibility scores between the two groups before and after intervention is shown in Table 5.

**Table 5:** Comparison of cognitive flexibility scores between two groups of children with ASD before and after intervention (*M* ± SD).

Group	*N*	Pre-intervention	Post-intervention	*t*	*p*
Experimental	12	4.83 ± 1.53	5.67 ± 1.50	−2.803	0.017
Control	12	4.83 ± 1.75	5.42 ± 1.62	−1.343	0.206
*t*		0.000	0.392		
*p*		1.000	0.699		

#### 3.3.1 Between-group differences

Baseline before intervention: The cognitive flexibility score of the experimental group was 4.83 ± 1.53, and that of the control group was 4.83 ± 1.75. There was no significant between-group difference (*t* = 0.000, *p* = 1.000>0.05), with completely consistent baseline levels.

Post-intervention endpoint: The cognitive flexibility score of the experimental group increased to 5.67 ± 1.50, and that of the control group was 5.42 ± 1.62. The difference between the two groups was still not statistically significant (*t* = 0.392, *p* = 0.699>0.05), indicating that the between-group improvement effect of gymnastics exercises on cognitive flexibility was not significant in the short term.

#### 3.3.2 Within-group differences

Experimental group: The post-intervention cognitive flexibility score (5.67 ± 1.50) was significantly higher than the pre-intervention score (4.83 ± 1.53), with an average increase of 0.84 points and statistical significance (*t* = −2.803, *p* = 0.017<0.05), indicating that gymnastics exercises can promote the cognitive strategy switching ability of children in the experimental group.

Control group: The pre-intervention score was 4.83 ± 1.75, and the post-intervention score was 5.42 ± 1.62, with a small increase (average 0.59 points), but the difference was not statistically significant (*t* = −1.343, *p* = 0.206>0.05), suggesting that routine campus activities cannot effectively enhance the cognitive flexibility of children with ASD.

## 4 Discussion

This study systematically explored the improvement effects of 12-week structured gymnastics exercises on the three core components of executive function (inhibitory control, working memory, cognitive flexibility) in 6–9-year-old children with ASD through a RCT. The results showed that gymnastics exercises had positive effects on all three components, but there were dimensional differences in the effects. Meanwhile, gymnastics has unique advantages compared with other forms of exercise, and the study still has areas for improvement. The following is a detailed discussion combining existing research and the results of this study.

### 4.1 Improvement effect and mechanism of gymnastics exercises on inhibitory control

This study found that 12-week gymnastics exercises increased the inhibitory control score of the experimental group of children with ASD by an average of 1.83 points, with high statistical significance (*p* = 0.005 < 0.01), while the control group only increased by 0.59 points (*p* = 0.171 > 0.05), which is consistent with the conclusions of previous motor intervention studies. For example, some studies have found that 10-week children’s fun track and field can significantly improve the inhibitory control of children with ASD (Yan, 2023), and comprehensive exercise training can improve their attention and behavior regulation abilities (Zhao et al., 2021). This study further verifies the “targeted improvement effect of structured motor intervention on the inhibitory control of children with ASD,” and the effect of gymnastics is more clear in the within-group comparison.

From the perspective of potential neural mechanisms, gymnastics exercises may act on inhibitory control through multiple synergistic pathways: First, brain region function activation and enhanced connectivity. Gymnastics movements such as tumbling and balance beam turns require the collaborative participation of the prefrontal cortex (the core brain region of inhibitory control) and the cerebellum (the motor coordination center), and such complex movements can promote the functional connectivity of the prefrontal-parietal network (Davoudi et al., 2023). This has commonalities with the research mechanism of Cheng (2023) — the study found that 12-week youth basketball training can increase the thickness of the right anterior cingulate cortex in children with ASD, thereby improving working memory and inhibitory control, suggesting that “motor-prefrontal cortex remodeling” may be a common mechanism across exercise types. Second, regulation of neurotransmitters and neurotrophic factors. Exercise can promote the release of neurotransmitters such as dopamine and norepinephrine, among which dopamine D2 receptors are crucial for inhibitory control (Chen et al., 2011). Similarly, Liu (2023) found that youth basketball exercise improves the executive function of children with ASD by increasing the level of brain-derived neurotrophic factor (BDNF), and the whole-body, multi-joint coordination characteristics of gymnastics may more strongly activate the neuroendocrine system, indirectly promoting the release of such factors (although this study did not directly detect them, it can be inferred indirectly through behavioral effects). Third, improvement of sensory integration disorders. Movements such as crawling and directional jumping in gymnastics require the coordination of vision, proprioception, and vestibular sensation, which helps to alleviate the common sensory integration disorders in children with ASD (Liu et al., 2021). Shao (2014) also pointed out that basic gymnastics can enhance the body coordination and perception ability of young children with ASD, and the improvement of sensory integration can lay a foundation for the improvement of inhibitory control — when children can more clearly perceive the relationship between the body and the environment, it is easier to inhibit “impulsive behaviors caused by sensory overload” (such as unreasonable crying).

In addition, the movement complexity of gymnastics is the key to its advantages. Compared with single aerobic exercises such as jogging, the postural control during tumbling and the direction judgment during jumping place higher requirements on inhibitory control. Tse et al. (2019) also confirmed that complex sports such as basketball and baseball have better improvement effects on inhibitory control than jogging. The results of this study further support the view that “the higher the matching degree between movement complexity and cognitive demand, the more significant the improvement of inhibitory control”.

### 4.2 Improvement effect and mechanism of gymnastics exercises on working memory

The working memory score of the experimental group of children with ASD increased by an average of 11.33 points (*p* < 0.001), significantly higher than the 7.17 points of the control group (*p* = 0.013), and the between-group difference was close to statistical significance, indicating that the improvement effect of gymnastics exercises on working memory was better than that of routine campus activities. This result not only confirms the positive effect of motor intervention on cognitive function (He and Liang, 2013; Wang et al., 2023), but also highlights the unique value of gymnastics as a comprehensive sport. Its improvement mechanism can be analyzed from three aspects: First, remodeling of brain structure and function. Movements such as balance beam turns and multi-directional crawling in gymnastics depend on the collaborative operation of the prefrontal cortex (the core brain region of working memory) and the cerebellum — children need to monitor body posture in real time and adjust movement amplitude to adapt to environmental changes (such as avoiding obstacles), and this continuous neural activation can enhance the functional connectivity between brain regions and optimize information processing efficiency (Zhu, 2020). Wang (2020) and Cheng (2023) also found similar mechanisms in youth basketball intervention: 12-week training can change the local consistency of the prefrontal cortex in children with ASD, thereby improving executive function. It can be seen that “repeated activation of the prefrontal-cerebellar network to promote brain function remodeling” is a common cognitive promotion path for gymnastics and ball games. Second, the regulatory role of neurotrophic factors. Exercise can stimulate the secretion of BDNF, and as a key regulatory factor of neural plasticity, BDNF can promote neuron survival, synaptogenesis, and neural circuit remodeling, directly affecting the development of working memory-related brain regions (Sun et al., 2017). Liu (2023) confirmed that the BDNF level of children with ASD increased significantly after youth basketball exercise, and was positively correlated with the improvement of working memory. Compared with single ball games, gymnastics covers multiple types of movements such as walking, running, jumping, tumbling, and balancing, which requires the mobilization of the whole body muscles. Its whole-body movement characteristics may more strongly activate the neuroendocrine system, prompting an increase in the release of factors such as BDNF, providing a neural basis for the improvement of working memory. Third, integration of attention resources and exercise of the “refreshing function” of working memory. Gymnastics training requires children to process multi-modal information at the same time: visually, they need to observe movement demonstrations and route markers; aurally, they need to receive instructions and rhythm prompts; proprioceptively, they need to perceive body position and movement status. The simultaneous integration of such multi-sensory information forces the brain to complete the full process of “receiving-encoding-storing-extracting” within a limited time, significantly exercising the “refreshing function” of working memory (Liu et al., 2021). For example, in tumbling practice, children need to remember the movement sequence of “flexed body-tucked body-rolling-getting up,” while adjusting the force intensity according to the position of the mat. This process requires continuous updating of the movement representation in working memory to avoid errors caused by information overload. Long-term training can enhance the brain’s processing efficiency for complex information, making working memory more stable in multiple tasks.

The improvement of working memory in the control group may be related to simple cognitive stimulation in routine activities (such as rule understanding and command response in group games), but such activities lack systematic cognitive load design and are difficult to deeply activate the neural mechanisms related to working memory, so the effect is only 63.3% of that in the experimental group. This is consistent with the conclusions of Li (2022) and Zhang et al. (2025): comprehensive and structured motor interventions (such as gymnastics) have significantly better improvement effects on executive function than single and random physical activities.

### 4.3 Improvement effect and mechanism of gymnastics exercises on cognitive flexibility

The cognitive flexibility score of the experimental group of children with ASD increased significantly (*p* = 0.017 < 0.05), with an average increase of 0.84 points, while the control group only increased by 0.59 points (*p* = 0.206 > 0.05), indicating that gymnastics exercises can promote the cognitive strategy switching ability of children with ASD, echoing Li’s (2022) research conclusion that “comprehensive games improve the executive function of children with ASD”.

The improvement of cognitive flexibility mainly stems from the diversity and dynamics of gymnastics movements: from postural adjustment during tumbling (alternation of straight body side roll and flexed body side roll) to route planning during obstacle walking (continuous conversion of straight line→curve→obstacle), all require children to update movement strategies in real time — this process of “continuously adjusting rules to adapt to changes” can effectively activate the functional connectivity between the prefrontal cortex and the parietal lobe, strengthening the neural mechanism of cognitive switching (Cai and Zhang, 2019). Chen et al. (2024) further confirmed through functional near-infrared spectroscopy (fNIRS) technology that sports game intervention can enhance the neural activity of the prefrontal cortex in children with ASD, and the activation intensity of the dorsolateral prefrontal cortex is directly related to the improvement of cognitive flexibility. In this study, combined exercises of “crawling + tumbling + jumping” may improve the brain’s processing efficiency for rule switching through similar mechanisms.

It should be noted that the between-group difference in cognitive flexibility in this study did not reach statistical significance (*p* = 0.699 > 0.05), which may be due to two reasons: first, the intervention period was insufficient — Yan (2023) found that 10-week children’s fun track and field can significantly improve cognitive flexibility, and although the period of this study was longer (12 weeks), the stereotyped requirements of gymnastics movements (such as limb angles during tumbling) may lead to resistance in some children, weakening the intervention effect; second, the improvement of cognitive flexibility may have a “delayed effect,” which requires a longer time to appear in the between-group difference. However, the significant within-group improvement in the experimental group still indicates that gymnastics exercises have positive significance for the cognitive flexibility of most children with ASD.

### 4.4 Unique advantages of gymnastics exercises compared with other sports

Compared with common sports intervention forms such as ball games and track and field, gymnastics has “three-dimensional advantages” in improving executive function in children with ASD, specifically reflected in: First, movement diversity activating multiple sub-systems of executive function. The core feature of gymnastics is the “multidimensional complexity” of movements—not only covering basic patterns such as walking, running, jumping, tumbling, crawling, and balancing, but also deriving infinite combinations through variables such as direction (forward/lateral), speed (uniform/variable), and amplitude (large/small), which can precisely match different sub-systems of executive function: balance beam turning requires constructing dynamic representations of body spatial positions, directly training visual–spatial working memory; jumping exercises with password rhythms (e.g., “1-2-jump”) need to process verbal information and movement timing simultaneously, activating the phonological loop subsystem (Foxall et al., 2025); combined movements of tumbling and jumping require inhibiting “habitual movement tendencies” (e.g., jumping directly instead of tumbling first), strengthening inhibitory control. In contrast, the movement patterns of ball games (such as basketball and table tennis) are relatively single (mainly focusing on hand ball control and limb coordination), paying more attention to visual-motor coordination, and it is difficult to comprehensively cover multiple components of executive function. The “one technique, multiple exercises” characteristic of gymnastics enables children with ASD to simultaneously mobilize abilities such as spatial encoding, verbal storage, and information refreshing in a single training session, forming a synergistic improvement effect (Morsanuto et al., 2023).

Second, multisensory integration improving cognitive processing efficiency. Gymnastics has a “panoramic” feature in activating the sensory system: vision: tracking movement demonstrations and identifying route markers such as sign buckets; audition: capturing instructions such as “bend knees” and “turn around,” and perceiving the rhythm of slogans or whistles; proprioception: providing real-time feedback on core tightening during tumbling and landing balance during jumping. The simultaneous input and integration of such multi-modal information force the brain to complete the full process of “receiving-encoding-storing-extracting” within a limited time, significantly improving the information turnover efficiency of executive function (Dai, 2017). For example, in the combination of “obstacle crawling + tumbling,” children need to remember the visual route (S-shaped trajectory), auditory instructions (“tuck your body when approaching the finish line”), and proprioception (elbow support strength) at the same time, and the intensity of its cognitive challenge is much higher than the single sensory interaction of “passing-catching” in ball games. Long-term training can enhance the brain’s ability to filter and integrate multiple information, making executive function more stable in complex environments.

Third, emotional regulation strengthening cognitive efficacy through positive experiences. Gymnastics training provides children with ASD a “stepped achievement experience field”—from being unable to complete tumbling independently, to gradually mastering balance beam walking, and then to smoothly completing combined movements, each step of progress can bring a perceivable sense of success. This positive emotional experience has a “gain effect” on executive function: emotions such as pleasure and confidence can activate the reward circuit of the prefrontal cortex, enhance dopamine release, and then improve the information retention time of working memory and the response accuracy of inhibitory control (Jiang, 2017). Shao (2014) also observed similar phenomena: after young children with ASD independently completed gymnastics movements, the frequency of eye contact increased, stereotyped behaviors decreased, and emotional improvement indirectly promoted cognitive function improvement. Compared with ball games (which often cause anxiety due to competitiveness), the “individualized progression” design of gymnastics (each child can break through at their own pace) is more likely to create a safe and controllable training atmosphere, so that positive emotions and executive function form a virtuous circle, which is particularly important for children with ASD who have weak emotional regulation abilities.

### 4.5 Research limitations and future directions

Although this study provides an empirical basis for gymnastics intervention in executive function of children with ASD, there are still four limitations that need to be improved in follow-up studies: First, the sample size is small and the representativeness is limited. This study only included 24 children with ASD (12 in each group), and the overall sample size is small, which may reduce the statistical power (such as the between-group difference in cognitive flexibility not reaching significance); and the samples all come from a special school in Chenghua District, Chengdu, with regional homogeneity, making it difficult to fully generalize to children in other regions, different degrees of disability (such as severe ASD), or different educational placement forms (such as inclusive education in ordinary schools). Future studies need to expand the sample size (it is recommended that each group has at least 30 people), adopt multicenter recruitment, and ensure the balanced distribution of each subgroup (such as different ages and disability levels) to improve the universality of the conclusions.

Second, the assessment tools are single, making it difficult to comprehensively cover the dimensions of executive function. Inhibitory control is only assessed through the DNST, which focuses on “cognitive conflict inhibition” and cannot cover “response inhibition” (such as inhibiting immediate action impulses); working memory only uses the SOPT, focusing on visual-semantic working memory, and does not involve digital working memory (such as digit backward) and spatial working memory (such as Corsi block task) (Liang, 2014; Ren, 2021, 2022); cognitive flexibility is only assessed through the adapted WCST, and other classic tools such as “task switching paradigm” are not combined. Single tools may lead to “dimensional bias in the improvement of executive function.” Future studies should integrate multiple assessment paradigms (such as Go/No-go task to assess response inhibition and Flanker task to assess interference inhibition) to verify the intervention effect from different cognitive dimensions (Gao et al., 2025).

Third, the lack of long-term follow-up makes it impossible to verify the sustainability of the effect. This study only tracked the immediate effect of 12-week intervention, and did not set follow-up time points such as 3 months and 6 months, so it is impossible to clarify the persistence of the gymnastics intervention effect. The cognitive effect of motor intervention may have a “fading effect”—if continuous training is lacking, the executive function improved in the short term may decline over time. Follow-up longitudinal studies are needed to track the change trend for more than 1 year, and explore the “maintenance training program” (such as 1–2 advanced exercises per week) to provide a basis for the design of the intervention cycle.

Fourth, confounding variables are not controlled, and the exploration of neural mechanisms is insufficient. Confounding variables include not including variables such as nutritional status, family socioeconomic status, and parental rearing styles, which may indirectly affect the improvement of executive function by affecting children’s sports participation or neurodevelopmental environment; neural mechanisms: only observing effects at the behavioral level, without detecting physiological indicators such as BDNF levels and prefrontal cortex activity (such as fNIRS monitoring of blood oxygen changes) (Guo et al., 2025; Liu and Liu, 2017; Huang and Wang, 2020), so it is impossible to directly verify the hypothesis that “gymnastics improves executive function by remodeling brain function and regulating neurotrophic factors.” Future studies need to comprehensively control confounding variables and combine molecular biology and neuroimaging technologies to deepen the exploration of mechanisms and provide more precise scientific basis for the optimization of intervention programs.

### 4.6 Practical implications

Based on the results of this study, the application of gymnastics exercises in the rehabilitation of executive function in children with ASD can be promoted from four aspects: intervention design, home-school collaboration, teacher training, and clinical care: First, targeted intervention design focusing on movement complexity and adaptability. Core movement selection: preferably retain multi-sensory collaborative movements such as tumbling, balance beam walking, and obstacle crawling, and gradually increase cognitive load through the advanced mode of “basic movements→combined movements→contextual tasks” (such as from single tumbling to “tumbling + directional jumping” combination); intensity and frequency: maintain the intervention frequency of “3 times a week, 40 min each time,” and control the exercise intensity at a moderate level (average heart rate 100–120 beats/min) to avoid resistance caused by too high intensity.

Second, home-school collaboration to build a “school-led + family-continued” closed-loop. Family guidance: the school compiles the Home Simple Gymnastics Guide to guide parents to carry out adapted exercises such as kneeling and crawling (10 meters ×3 groups) and standing turns (5 circles on each side) for 15–20 min daily to continue the stimulation intensity of school intervention; parent–child interaction: enhance training compliance by “parents accompanying the completion of movements and jointly recording progress logs”; regularly organize parent workshops (once a month, 90 min each time) to train guidance skills such as “decomposed passwords + visual prompt cards” (such as simplifying tumbling instructions with “hold your chest like a ball and roll”) (Zhang and Deng, 2010).

Third, teacher training to strengthen the professional capabilities of special education teachers. Carry out special training on “gymnastics movement decomposition and personalized guidance,” and set up 3 core modules (total duration 24 h): basic module: principles of gymnastics movements (such as the core force points of tumbling), and movement adaptation skills for children with ASD (such as reducing the balance beam turn from 180° to 90°); emergency module: dealing with abnormal reactions such as vestibular hypersensitivity (such as dizziness and crying) and proprioceptive dullness (such as excessive movement amplitude), and training “pause signals (holding a red card)” and “alternative movements (side lying rolling instead of forward rolling)”; feedback module: introduce video review technology, extract 1 training video every week for collective analysis, and correct the guidance method to ensure intervention fidelity (Guo, 2022).

Fourth, clinical care integrating multi-scene gymnastics intervention. In the clinical care of children with ASD, gymnastics can be used as a low-cost non-drug intervention method for scene-based application: family: adopt “fragmented training” (morning tumbling in bed, tiptoeing to get objects before meals) + “structured training” (3 times a week of advanced exercises on gymnastics mats); school: organize “movement relay races” (drilling through desk holes→single-leg jumping→clapping and turning) during breaks, and group physical education classes by ability (basic group balance training, advanced group vaulting box exercises); psychiatric clinic: carry out 10-min “movement desensitization training” (walking straight with eyes closed→walking around obstacles with eyes open) before individual therapy to help children transition from restlessness to focus; carry out “mirror imitation games” in group therapy to train inhibitory control and social interaction simultaneously.

The improvement of executive function is of key significance for the clinical care of children with ASD—it can not only reduce impulsive behaviors (such as interrupting conversations for no reason) and emotional problems (such as self-harm), prolong the time to follow rules in group activities, but also promote the acquisition of social skills (such as flexible interaction in role-playing), laying a foundation for their integration into general education environments, reducing family care burdens, and independent living in adulthood.

## 5 Conclusion

This study systematically explored the intervention effects of 12-week structured gymnastics exercises on the three core components of executive function in 6–9-year-old children with ASD through a RCT. The results showed that 12-week gymnastics exercises had an overall positive improvement effect on the executive function of children with ASD: In terms of inhibitory control, the post-intervention score of the experimental group significantly increased compared with the pre-test, while the control group showed no significant change. The mechanism was related to gymnastics movements activating the prefrontal-cerebellar network and improving sensory integration disorders. In terms of working memory, the score of the experimental group increased compared with the pre-test, with an improvement range 1.58 times that of the control group, and the between-group difference was close to significance. The improvement was achieved through brain function remodeling, neurotrophic factor regulation, and attention integration. In terms of cognitive flexibility, the score of the experimental group significantly increased compared with the pre-test, with walking/running and tumbling movements showing prominent effects, while the control group had no significant change, attributed to the diversity of gymnastics movements strengthening the functional connectivity of the prefrontal-parietal network.

Although this study provides an empirical basis for gymnastics intervention, there are still limitations: only 24 children with ASD from a single special school were included, with insufficient sample representativeness and stability; inhibitory control, working memory, and cognitive flexibility were assessed only through the DNST, SOPT, and adapted WCST, respectively, with single tools that were difficult to cover the multiple characteristics of each dimension; only the immediate effect of 12 weeks was tracked, and no 3–6-month follow-up was set to verify the sustainability of the effect; confounding variables such as nutrition and family socioeconomic status were not included, and physiological indicators such as BDNF and prefrontal activity were not detected, making it impossible to directly verify the neural mechanism hypothesis.

Future research needs to improve representativeness by expanding the sample size and adopting multicenter recruitment, deepen mechanism exploration by combining multiple tools such as Go/No-go tasks and digit span tests with fNIRS and BDNF detection, track 1-year follow-up and design maintenance training programs, and control confounding variables to enhance the rigor of conclusions. In practice, gymnastics can be incorporated into special education curricula (3 times a week, 40 min each time, strengthening tumbling and balancing movements), a home-school collaborative intervention closed-loop can be constructed through the Home Simple Gymnastics Guide, and policy support can be relied on to increase facility investment, train full-time teachers, and include it in rehabilitation subsidies, so as to promote the standardized implementation of gymnastics intervention, provide safe and easy-to-operate non-drug cognitive rehabilitation programs for 6–9-year-old children with ASD, and maximize its value in supporting social adaptation.

## Data Availability

The original contributions presented in the study are included in the article/supplementary material, further inquiries can be directed to the corresponding author/s.

## References

[ref1] American Psychiatric Association (2013). Diagnostic and statistical manual of mental disorders. 5th Edn. Washington, DC: American Psychiatric Publishing.

[ref2] An W. J. Hu S. S. Guo W. Wu X. Y. Wang H. P. (2019a). Advances of research on working memory of children and teenagers with autism spectrum disorders. Chinese J Spec Educ 8, 56–62.

[ref3] An W. J. Li J. H. Wang Y. F. Wu X. Y. Wang H. P. (2019b). Research progress in inhibitory control in children and adolescents with autism spectrum disorders. Chinese J Spec Educ 4, 32–39.

[ref4] Arango-Lasprilla J. C. Rivera D. Nicholls E. Arelis A. A. de la Cadena C. G. Guia A. I. P.. (2017). Modified Wisconsin card sorting test (M-WCST): normative data for Spanish-speaking pediatric population. Neurorehabil. 41, 1–10. doi: 10.3233/NRE-172242 28946592

[ref6] Cai C. X. Zhang Y. L. (2019). Research progress on the mechanism of exercise improving brain executive function. J Chengdu Sport Univ 45, 120–126. doi: 10.15942/j.jcsu.2019.06.020

[ref7] Chen H. Liang Q. Wang B. J. Liu H. X. Dong G. J. Li K. F. (2024). Sports game intervention aids executive function enhancement in children with autism-An fNIRS study. Neurosci. Lett. 822:137647. doi: 10.1016/j.neulet.2024.137647 38242348

[ref8] Chen A. G. Yin H. C. Yan J. Yang Y. (2011). Effects of acute aerobic exercise of different intensity on executive function. Acta Psychol. Sin. 43, 1055–1062.

[ref9] Cheng W. (2023). Mini basketball teaching program improves executive function and brain cortical thickness in preschool children with autism. Yangzhou: Yangzhou University.

[ref10] Crawley D. Zhang L. Jones E. J. H. Ahmad J. Oakley B. Cáceres A. S.. (2020). Modeling flexible behavior in childhood to adulthood shows age-dependent learning mechanisms and less optimal learning in autism in each age group. PLoS Biol. 18:e3000908. doi: 10.1371/journal.pbio.3000908 33108370 PMC7591042

[ref11] Dai W. X. (2017). An experimental study of children with autism spectrum in regular class for bridging inference processing in discourse reading. Shanghai: East China Normal University. 1:11.

[ref12] Davoudi S. Rahdar M. Hosseinmardi N. Behzadi G. Janahmadi M. (2023). Chronic inhibition of astrocytic aquaporin-4 induces autistic-like behavior in control rat offspring similar to maternal exposure to valproic acid. Physiol Behav 269:114286. doi: 10.1016/j.physbeh.2023.114286, PMID: 37402416

[ref13] Demetriou E. A. Lampit A. Quintana D. S. Naismith S. L. Song Y. J. C. Pye J. E.. (2018). Autism spectrum disorders: a meta-analysis of executive function. Mol Psychiatry 23, 1198–1204. doi: 10.1038/mp.2017.75, PMID: 28439105 PMC5984099

[ref14] Diamond A. (2013). Executive functions. Annu Rev Psychol 64, 135–168. doi: 10.1146/annurev-psych-113011-143750, PMID: 23020641 PMC4084861

[ref15] Faja S. Darling L. N. (2019). Variation in restricted and repetitive behaviors and interests relates to inhibitory control and shifting in children with autism spectrum disorder. Autism 23, 1262–1272. doi: 10.1177/1362361318804192, PMID: 30394786 PMC6499722

[ref16] Foxall V. Leckey J. Brandt A. Jacques S. Johnson S. (2025). The effects and characteristics of exercise interventions on executive functioning in individuals with autism spectrum disorder: a scoping review. Rev J Autism Dev Disord 2025, 1–29. doi: 10.1007/s40489-025-00510-4

[ref17] Gao S. Q. Yi Y. Liancheng Z. Chenli Y. (2025). Promoting ellect of exercise-inteerated coenitive intervention on the inhibition control function of colege students. Chinese J school. Health 46, 703–707. doi: 10.16835/j.cnki.1000-9817.2025116

[ref1001] Gesundheit B. Hochbaum L. Fetyukhina A. Vorobyev V. Vorobyev N. (2025). Mesenchymal stromal cell treatment alleviates autism spectrum disorder symptoms: a case report. Cureus 17:e83440. doi: 10.7759/cureus.83440 40462775 PMC12130910

[ref18] Gomez-Pinilla F. Hillman C. (2013). The influence of exercise on cognitive abilities. Compr Physiol 3, 403–428. doi: 10.1002/cphy.c110063 23720292 PMC3951958

[ref19] Guo Y. (2022). Study on the design of individualized education curriculum for autistic children in PE classes. Xi’an Phys Educ Univ. doi: 10.27401/d.cnki.gxatc.2022.000045

[ref20] Guo Y. Zhang L. C. Tao Y. Y. Zhu L. H. Wang T. (2025). Is BDNF an underlying biological mechanism in exercise-induced cognition? Evidence, challenges, and prospects. Adv Psychol Sci 33, 465–476.

[ref21] He H. Z. Liang Z. G. (2013). A review on the executive function of children with autism. Theory Pract Educ 33, 45–48.

[ref22] Hillman C. H. Mcdonald K. M. Logan N. E. (2020). A review of the effects of physical activity on cognition and brain health across children and adolescence. Nestle Nutr Inst Workshop Ser 95, 116–126. doi: 10.1159/000511508, PMID: 33161407

[ref23] Hillman C. H. Pontifex M. B. Castelli D. M. Khan N. A. Raine L. B. Scudder M. R.. (2014). Effects of the FITKids randomized controlled trial on executive control and brain function. Pediatrics 134, e1063–e1071. doi: 10.1542/peds.2013-3219, PMID: 25266425 PMC4179093

[ref24] Hou T. T. Yang F. Y. (2016). A review of the research into the executive functions of preschool children with autism. Chinese J Spec Educ 3, 10–16.

[ref25] Huang J. Wang T. (2020). The neural mechanism of social cognitive deficits in autism spectrum disorders: evidence from fNIRS. Chin J Spec Educ 6, 32–38.

[ref26] Jiang J. (2017). A study on exploring the ability of lyingin children with ASD and its affecting factors. Hangzhou: Hangzhou Normal University.

[ref27] Jing S. G. (2001). On the feasibility of rolling and up-side-down movement composed in national standard of physical exercising. J Hebei Sport Univ 2, 29–31.

[ref28] Kelly S. E. Schmitt L. M. Sweeney J. A. Mosconi M. W. (2021). Reduced proactive control processes associated with behavioral response inhibition in autism spectrum disorder. Autism Res 14, 389–399. doi: 10.1002/aur.2415 33111461 PMC7878417

[ref29] Kou L. Fan L. Y. (2020). Intervention effect and influencing factors of cheerleading on young autistic children. Occup Health 36, 474–477+481. doi: 10.13329/j.cnki.zyyjk.2020.0128

[ref1002] Li H. Gao S. Wang N. Y. (2004). A review on research methods of executive function. Adv Psych Sci 5, 693–705.

[ref30] Li M. X. (2014). A summary of the researches for the working memory in autistic children. J Huzhou Univ 36, 81–85.

[ref31] Li Y. (2022). Effects of different game intervention on the executive function of children with autism. Ürümqi: Xinjiang Normal University.

[ref32] Li Y. X. (2022). Ball games combined with sports games on preschool autism effect of executive function in children. Taiyuan: Shanxi University.

[ref33] Li D. Y. Zhang L. W. (2020). The improvement effects of natural environments on inhibitory and persistent self-control in cognitive and motor tasks. China Sport Sci Technol 56, 31–44. doi: 10.16470/j.csst.2019037

[ref34] Liang Z. G. (2014). Relationships between cognitive flexibility, repetitive behaviors for children with autism. Shanghai: East China Normal University.

[ref35] Liu S. S. (2023). Cold and hot executive functions characteristics and intervention study with ASD children. Jinan: Jinan University.

[ref36] Liu Y. X. (2023). Mini-basketball training program improves executivefunction and brain derived neurotrophic factor inschool-age children with autism spectrum disorders. Yangzhou: Yangzhou University.

[ref37] Liu J. (2025). *Decoding the “golden intervention period” of autism*. Harbin Daily.

[ref38] Liu Z. T. He Y. Q. Huang C. X. (2021). Progress in the application of exercise intervention in autistic children. J Int Psych 48, 961–964+970. doi: 10.13479/j.cnki.jip.2021.06.001

[ref39] Liu T. Liu X. C. (2017). Functional near-infrared spectroscopy studies on social deficits in children with autism. J Psychol Sci 40, 1005–1010. doi: 10.16719/j.cnki.1671-6981.20170434

[ref40] Lord C. Elsabbagh M. Baird G. Veenstra-Vanderweele J. (2018). Autism spectrum disorder. Lancet 392, 508–520. doi: 10.1016/S0140-6736(18)31129-2, PMID: 30078460 PMC7398158

[ref41] Lu M. Yang G. Skora E. Wang G. Cai Y. Sun Q.. (2015). Self-esteem, social support, and life satisfaction in Chinese parents of children with autism spectrum disorder. Res Autism Spectr Disord 17, 70–77. doi: 10.1016/j.rasd.2015.05.003

[ref42] Ministry of Education of the People’s Republic of China (2018). Curriculum standards for compulsory education in life adaptation in schools for intellectually disabled students. Beijing: People's Education Press.

[ref43] Montgomery D. E. Koeltzow T. E. (2010). A review of the day–night task: the Stroop paradigm and interference control in young children. Dev Rev 30, 308–330. doi: 10.1016/j.dr.2010.07.001

[ref44] Morsanuto S. Cassese F. P. Tafuri F. Tafuri D. (2023). Outdoor education, integrated soccer activities, and learning in children with autism spectrum disorder: a project aimed at achieving the sustainable development goals of the 2030 agenda. Sustainability 15, 1–14. doi: 10.3390/su151813456

[ref45] Ozonoff S. Strayer D. L. (1997). Inhibitory function in nonretarded children with autism. J Autism Dev Disord 27, 59–77. doi: 10.1023/A:1025821222046, PMID: 9018582

[ref46] Ren G. F. (2021). An experimental research on sentence comprehension of children with high functioning autism. Shanghai: East China Normal University.

[ref47] Ren Z. Q. (2022). A study on the processing of the deictic usages of “zhe” and “na” in Chinese high-functioning autistic children. Nanjing: Nanjing Normal University.

[ref48] Sallum I. da Mata F. G. Cheib N. F. Mathias C. W. Miranda D. M. Malloy-Diniz L. F. (2017). Development of a version of the self-ordered pointing task: a working memory task for Brazilian preschoolers. Clin Neuropsychol 31, 459–470. doi: 10.1080/13854046.2016.1275818, PMID: 28079460

[ref49] Shao Q. (2014). A case study on the rehabilitation effect of basic gymnastics on children with autism aged 3-6 years old. Mod Educ 5, 55–57.

[ref50] Sun Y. Fang L. Wang T. Y. Cui L. (2018). The influence factors and neural mechanisms of inhibitory control in autism spectrum disorders. Adv Psychol Sci 26, 1450–1464.

[ref51] Sun L. N. Sun Q. S. Qi J. S. (2017). Adult hippocampal neurogenesis: an important target associated with antidepressant effects of exercise. Rev Neurosci 28, 693–703. doi: 10.1515/revneuro-2016-0076, PMID: 28422706

[ref52] Tao G. T. (1982). The diagnosis and attribution of infantile autism. Chin J Neurol 15, 104–107. 7128306

[ref53] Tse C. Y. A. Lee H. P. Chan K. S. K. Edgar V. B. Wilkinson-Smith A. Lai W. H. E. (2019). Examining the impact of physical activity on sleep quality and executive functions in children with autism spectrum disorder: a randomized controlled trial. Autism 23, 1699–1710. doi: 10.1177/1362361318823910, PMID: 30663324

[ref54] Wang J. G. (2020). Mini-basketball training program improves executive function and regional homogeneity in preschool children with autism spectrum disorders. Yangzhou: Yangzhou University.

[ref55] Wang Y. He G. Li D. S. Ma K. Y. Wang C. (2023). Effect of physical activity on executive function of children and adolescents: a systematic review. Chin J Rehabil Theory Pract 29, 20–29.

[ref56] Wen Y. (2012). Gymnastics tutorial for young children. Beijing: People’s Education Press.

[ref57] Wucailu Autism Research Institute (2024). Development report on the autism education and rehabilitation industry in China (V). Beijing: Guangming Publ House.

[ref58] Yan K. K. (2023). The influence of kid athletics on the physical health and executive function of autistic children. Taiyuan: Shanxi University.

[ref59] Yang W. (2016). *The role of happy gymnastics in the physical and mental development of preschool children*. General Administration of Sport of China Official Website, 2016-04-25. Available online at: https://www.sport.gov.cn/n20001280/n20067626/n20067861/c20212042/content.html.

[ref60] Zhang L. (2023). A study on the effects of multi-directional mobile games on motor coordination and executive function of 5-6 years old children. Jinan: Shandong Sport University.

[ref61] Zhang Z. Y. Deng S. H. (2010). Case study on sports games intervention of autistic children. China Sport Sci 30, 49–56+68. doi: 10.16469/j.css.2010.08.004

[ref62] Zhang Y. H. Tian H. D. Tao Y. H. Li Y. Wang D. R. Qin L. X. (2025). A study on the effects of three game intervention programs on executive functions of preschool autistic children. Int J Dev Disabil 71, 168–178. doi: 10.1080/20473869.2023.2215606 39882406 PMC11774177

[ref63] Zhao F. Lu F. Tan C. H. Shu D. M. Ma S. S. Liu D. Z. (2021). Effects of graded comprehensive exercise intervention on children with autism spectrum disorder——comparison of ADHD comorbidities and non-comorbidities. Chin J Clin Psychol 29, 869–875. doi: 10.16128/j.cnki.1005-3611.2021.04.042

[ref64] Zhu S. Y. (2020). Research on characteristics of the executive control network and the temporal dynamic spontaneous brain activities in children with autistic spectrum disorder. Shanghai: Shanghai Jiao Tong University.

